# Alcohol misuse, drinking contexts and intimate partner violence in St. Petersburg, Russia: results from a cross-sectional study

**DOI:** 10.1186/1471-2458-11-629

**Published:** 2011-08-05

**Authors:** Weihai Zhan, Alla V Shaboltas, Roman V Skochilov, Andrei P Kozlov, Tatiana V Krasnoselskikh, Nadia Abdala

**Affiliations:** 1 Yale School of Public Health, 60 College Street, New Haven, CT, 06520, USA; 2 The Biomedical Center, 8 Vyborgskaya ul, St. Petersburg, 194044, Russian Federation; 3 Saint Petersburg, State University, 7/9 Universitetskaya nab, St Petersburg, 199034, Russian Federation; 4 Pavlov State Medical University, 6/8 Leo Tolstoy Str., St Petersburg, 197022, Russian Federation

## Abstract

**Background:**

Alcohol misuse has been linked to intimate partner violence (IPV). However, this association is not usually examined in Russia. Moreover, more investigation is required as to whether specific drinking contexts are also associated with IPV. The objectives of this study are: to investigate whether alcohol misuse is associated with IPV and to further examine whether specific drinking contexts among drinkers are associated with IPV.

**Methods:**

A questionnaire was used to collect information on demographics, health status, alcohol use, and violence involving sexual partners among 440 participants who were recruited from an STI (sexually transmitted infection) clinic center in St. Petersburg, Russia for a cross-sectional study from 2008 to 2009. Multivariate logistic regression was used for analysis.

**Results:**

Overall, 47.0% participants were classified as misusing alcohol and 7.2% participants perpetrated IPV in the past three months. Participants with alcohol misuse were 3.28 times (OR: 3.28; 95% CI: 1.34-8.04) as likely as those without alcohol misuse to perpetrate IPV. Among participants who had consumed alcohol in the past three months, those who usually drank on the streets or in parks (OR: 5.62; 95% CI: 1.67-18.90) were more likely to perpetrate IPV.

**Conclusions:**

Both alcohol misuse and certain drinking contexts (e.g., drinking on the streets or at parks) were associated with IPV. The association between drinking contexts and IPV needs further investigation, as do the underlying mechanisms for this association. IPV prevention initiatives might benefit from reducing alcohol misuse. Drinking contexts such as drinking on the streets or at parks as well as the factors related to the use of alcohol in these contexts may also need to be addressed.

## Background

Alcohol consumption, particularly at hazardous drinking levels, is highly prevalent in Russia. The estimates based on 2001-2003 data revealed that, on average, each Russian aged 15 years and older consumed 15.2 L of pure ethanol alcohol per year, among the highest rates in the world [1]. Another study conducted in a western city of Russia between 1999-2000 showed that 75% of male and 47% of female workers were classified as misusing alcohol according to the Alcohol Use Disorder Identification Test (AUDIT) criteria [2]. Alcohol misuse causes serious public health problems in Russia. It has been reported that more than half of all deaths at ages 15-54 years in Russia were alcohol related, during the period from 1990 to 2001 [3].

Alcohol misuse has also been linked to intimate partner violence (IPV) in both experimental and empirical studies [4,5]. In a multicenter case-control study conducted at eight university-affiliated emergency departments in the United States, male partners who abused alcohol were 3.6 times (95% confidence interval [CI], 2.2-5.9) more likely to perpetrate IPV compared to those without alcohol abuse issues [5]. Despite no consensus having been reached, several theoretical models have been proposed to explain this relationship [4]. As a more evidence-based model, the proximal effects model suggests that alcohol misuse causes IPV through psychopharmacological effects [4]. Briefly, alcohol misuse may distort perceptions of cues and reduce abilities of self-inhibition, which, consequently makes people more likely to solve problems in a violent way [6]. In addition to the direct psychopharmacological effects, alcohol misuse may also have indirect effects on IPV, such as through increasing conflicts and dissatisfaction in family/partnership life [7].

A limited number of studies on IPV in Russia demonstrated that IPV is common in the country [8-10]. A survey conducted among 3,900 women from three Russian cities found that nearly 15% of women had been moderately or severely abused by their partners [8]. Although a small number of IPV studies in Russia focused on men's IPV against women, both men and women can perpetrate IPV against their partners [10]. In a study conducted in three Russian universities, 22.8% of male and 37.6% of female students had physically assaulted their partners; 24.4% of male and 22.9% of female students had intended to compel the partner to engage in unwanted sexual activity [10].

The association between alcohol misuse and IPV is much less investigated in Russia, particularly in comparison to countries in North America and Western Europe [11]. Moreover, some evidence indicates that drinking contexts may play an important role in the association between alcohol misuse and violence [12,13]. For example, in one study, a higher frequency of visits to public drinking places was significantly associated with a higher probability of having been in a fight under the influence of alcohol [13]. Another study found that couples with discrepant drinking patterns were more likely to experience IPV than those sharing similar drinking habits [12].

The examination of drinking context and its association with IPV in Russia is also important because it may provide valuable information for IPV intervention programs. Thus, we conducted a study to investigate whether alcohol misuse is associated with IPV among participants from a sexually transmitted infection (STI) clinic in St. Petersburg, Russia. As a secondary aim, we examined whether certain types of drinking settings are associated with IPV among alcohol users.

## Methods

### Study participants and data collection

From June 2008 to June 2009, a cross-sectional study was conducted among patients who visited a public STI clinic in St. Petersburg, Russia. The city of St. Petersburg is comprised of 18 districts, nearly all containing a public STI outpatient clinic (i.e., a district dispensary for skin and venereal diseases). The current study was conducted at the genitourinary department of the Kalininsky clinic. The clinic provides services free of charge or for a nominal fee to patients, the majority of whom are local residents. Consecutive adult patients aged 18 years and older who reported genitourinary complaints or had a need for STI-related services (e.g., STI testing or concern about STIs) were invited to participate in the study. In total, 500 patients were eligible; 440 of them agreed to participate and completed a self-administered questionnaire. The study was approved by the institutional review boards of the Biomedical Center in St. Petersburg, Russia and Yale University in Connecticut, USA.

The questionnaire contained 58 items and took about 15 minutes to complete. Demographics, alcohol use and behaviors towards sex partners were collected in the questionnaire. Demographic variables included age, sex, education, employment status, marital status and monthly income. Alcohol use information included frequency of alcohol use, frequency of having six or more drinks on one occasion as well as drinking contexts (i.e. preferred types of alcoholic beverages, preferred locations for drinking and preferred persons to drink with). Behaviors towards sex partners included history (lifetime and in the past three months) of insulting, swearing at or threatening a sexual partner, history of pushing, grabbing, slapping, punching, beating up or choking a sexual partner, and history of physically forcing a sexual partner to have sex or forcing a sexual partner to do something sexually that he/she did not want to.

### Measures

#### Alcohol misuse

Using a modified version of the three-question based Alcohol Use Disorders Identification Test (AUDIT-C), participants were asked the following three questions:

1) How often have you had a drink containing alcohol in the past three months?

2) On average, how many servings of drinks containing alcohol do you typically consume on a day when you are drinking?

3) How often, in the past three months, did you have six or more drinks on one occasion?

Each option for each question has been allotted a score between zero and four and thus the score range of AUDIT-C is between zero and 12. A score of four or more in men and three or more in women were considered positive (alcohol misuse). AUDIT-C has been widely accepted as a practical and valid screening test for alcohol misuse [14,15].

Participants were also asked three drinking context questions. The three questions and responses were as follows: "Which alcohol beverages do you usually drink (you may choose several options)?" The possible responses were beer, gin and tonic or other alcoholic cocktails, wine/champagne, liquor/sherry/vermouth, vodka, brandy and other strong beverages, and other. "At what venue do you usually drink (you may choose several options)?" The possible responses were home, bar, restaurant, night club, friend's place (party), street/park, staircase, other, and anywhere. "With whom do you drink most often?" The possible responses were friends, spouse, parents or other relatives, colleagues, sexual partner(s), alone, other, and with anyone.

#### Violence

The measurement of IPV was based on the revised version of the Conflict Tactics Scale [16]. In the present study, IPV was defined as ever having insulted, sworn at, threatened, pushed, grabbed, slapped, punched, beaten, choked, or physically forced a partner to engage in sex or forced a sexual partner to do something sexually that he/she did not want to do, within the past three months. To better understand the association between alcohol misuse and IPV, we chose IPV occurring within the past three months rather than lifetime IPV as our primary IPV definition because alcohol consumption was measured during the same time frame.

### Statistical analysis

Logistic regression models were used to determine if alcohol misuse was associated with IPV among all participants. Among the subgroup of participants who had drunk alcohol in the past three months, we examined whether drinking contexts were associated with IPV. If two or more drinking contexts were found to be significantly associated with IPV, these variables were later included in the same model to determine if each drinking context might still have an independent effect on IPV. The significance level was defined as p < 0.05, and SAS software (version 9.1, SAS Institute Inc., Cary, NC) was used to analyze the data.

## Results

### Characteristics and behaviors

Among the 440 patients who participated in the study, 286 (65.0%) were men and 154 (35.0%) were women (Table 1). The mean and median ages for participants were 28.4 and 25.0 years, respectively. A majority of the subjects were employed full time (61.6%) and had completed college education (72.1%). About 41.6% of the participants had a monthly income of less than 15,000 rubles (about 530 US dollars). The alcohol misuse rate was 47.0%. The overall prevalence of IPV perpetration was 7.2% and there was no gender difference in IPV prevalence.

**Table 1:** Characteristics of participants in St. Petersburg, Russia (N = 440)

Characteristics	Proportion
Age (25 years or less)^a^	233/438 (53.2%)
Being male	286/440 (65.0%)
Being married	192/439 (43.7%)
Completed college or more	317/440 (72.1%)
Monthly income < 15,000 rubles	170/409 (41.6%)
Full time employment	271/440 (61.6%)
Alcohol misuse	198/421 (47.0%)
Intimate partner violence^b^	30/414 (7.2%)

^a^The mean and median ages for participants were 28.4 and 25.0 years, respectively.

^b^There was no gender difference in IPV prevalence

### Alcohol misuse and IPV (controlling for socio-demographics)

In the bivariate analysis, marital status, education, monthly income and alcohol misuse were significantly associated with IPV (Table 2), whereas age, gender and employment status were not significantly associated with IPV. In the multivariate analysis, participants who misused alcohol were 3.28 times (OR, 3.28; 95% CI, 1.34-8.04) as likely to perpetrate IPV as those who did not misuse alcohol, adjusting for significant covariates (including marital status, education and monthly income).

**Table 2:** Association between alcohol misuse and intimate partner violence in St. Petersburg, Russia, controlling for socio-demographics (N = 440)

Characteristics	Unadjusted OR	Adjusted OR
Age (25 years or less)	0.70 (0.33-1.49)	-
Being male	0.84 (0.39-1.80)	-
Being married	2.73 (1.24-5.99)	3.29 (1.36-7.97)
Completed college or more	0.41 (0.19-0.87)	0.42 (0.18-0.99)
Monthly income < 15,000 rubles	2.49 (1.11-5.58)	2.56 (1.07-6.13)
Full time employment	0.59 (0.28-1.25)	-
Alcohol misuse	2.75 (1.22-6.20)	3.28 (1.34-8.04)

### Drinking context and IPV

Of the 440 participants, 373 drank alcohol in the past three months (Table 3). In general, participants usually drank beer (52.5%) and strong alcoholic beverages (44.8%), such as vodka and brandy. Participants usually drank alcohol at a friend's place, home and/or bar. However, about 12% participants usually drank alcohol in the street or park. Nearly 90% of the participants usually drank alcohol with their friends. The proportions of participants usually drinking with a spouse or sex partner(s) were 17.4% and 26.1%, respectively.

**Table 3:** Proportion of participants with different drinking contexts and association between drinking contexts and IPV among drinkers in St. Petersburg, Russia (N = 373)

Drinking Contexts	Proportion	Unadjusted OR (95% CI)	Adjusted OR (95% CI)^a^
Type of alcohol which the participant usually drank^b^			
Beer	193/368 (52.5%)	0.85 (0.38-1.92)	1.88 (0.68-5.21)
Alcoholic cocktails	29/368 (7.9%)	3.09 (1.07-8.97)	2.17 (0.60-7.88)
Wine	137/368 (37.2%)	0.79 (0.33-1.88)	0.85 (0.31-2.35)
Liquor/sherry/vermouth	40/368 (10.9%)	2.85 (1.06-7.66)	4.19 (1.34-13.15)
Strong alcoholic beverages (e.g., vodka and brandy)	165/368 (44.8%)	1.40 (0.62-3.16)	1.25 (0.48-3.26)
Venue where the participant usually drank^b^			
Home	200/358 (55.9%)	0.86 (0.38-1.94)	1.37 (0.52-3.61)
Bar	151/358 (42.2%)	1.15 (0.50-2.60)	1.39 (0.52-3.77)
Restaurant	99/357 (27.7%)	1.02 (0.41-2.52)	1.74 (0.63-4.83)
Night club	96/358 (26.8%)	1.34 (0.56-3.23)	1.49 (0.53-4.19)
Friend's place (party)	218/358 (60.9%)	0.70 (0.31-1.57)	0.81 (0.30-2.14)
Street or park	43/358 (12.0%)	3.53 (1.37-9.10)	5.58 (1.80-17.30)
Person with whom participant drank most often^b^			
Friends	320/357 (89.6%)	0.60 (0.19-1.86)	0.85 (0.16-4.43)
Spouse	62/357 (17.4%)	1.22 (0.44-3.39)	0.87 (0.24-3.13)
Parents or other relatives	68/357 (19.1%)	1.41 (0.54-3.68)	1.28 (0.38-4.28)
Colleagues	87/357 (24.4%)	1.29 (0.52-3.21)	1.58 (0.55-4.57)
Sex partner(s)	93/357 (26.1%)	1.43 (0.59-3.44)	2.82 (0.96-8.30)
Alone	16/357 (4.5%)	1.04 (0.13-8.33)	0.93 (0.10-8.37)

^a^Adjusting for alcohol misuse, marriage status and monthly income; education was not independently significantly associated with IPV in this sub-group.

^b^The added number of each category may exceed the total number of drinkers because participants were allowed to choose multiple options.

We then examined the associations between drinking contexts and IPV among drinkers. In two separate models, we found that the preference for drinking liquor/sherry/vermouth (odds ratio [OR]: 4.19, 95% confidence intervals [CI]: 1.34-13.15) and drinking in the street or park (OR: 5.58, 95% CI: 1.80-17.30) were significantly associated with IPV. These ORs were adjusted for significant covariates including alcohol misuse, marriage status and monthly income. Age, gender, education and employment status were not independently significantly associated with IPV among drinkers.

Considering that these two drinking context variables (drinking liquor/sherry/vermouth and drinking in the street or park) may confound each other, we examined their associations with IPV in the same model, further adjusting for AUDIT-C, marriage status and monthly income (Table 4). We found that the association between drinking in the street or park and IPV remained significant (OR, 5.62, 95% CI, 1.67-18.90), while the association between drinking liquor/sherry/vermouth and IPV was no longer significant (OR, 3.06, 95% CI, 0.90-10.41).

**Table 4:** Associations between drinking contexts and intimate partner violence among participants who used alcohol in the past three months in St. Petersburg, Russia (N = 373)

Potential triggers	Adjusted OR^a^
Prefer liquor/sherry/vermouth	3.06 (0.90-10.41)
Usually drinking at street or park	5.62 (1.67-18.90)
Being married	6.23 (1.99-19.49)
Monthly income < 15,000 rubles	5.55 (1.91-16.13)
Alcohol misuse	6.35 (1.90-21.28)

^a^Other socio-demographic variables, including age, sex, education and employment status, were not significantly associated with intimate partner violence and thus were not included in the model.

## Discussion

Although violence against women has been pervasive for a long time in Russian culture [9], controversy emerges over whether women are as violent as men in Russia as well as in other countries [10,17]. The present study provides new evidence that Russian women had a similar IPV perpetration rate as Russian men, suggesting that violence against men should not be neglected. A meta-analytic review of 82 IPV studies even shows that women were slightly more likely than men to perpetrate IPV, despite that women were more likely to be injured [18]. It is important to note that a majority of studies in this review were carried out in the United States. Further research considering gender role on IPV in Russia is still needed.

In the present study, we found that alcohol misuse was significantly associated with IPV among patients attending an STI clinic in St. Petersburg, Russia, which is consistent with results from previous studies conducted in Russia [11,19,20] and elsewhere [4,5,7,21,22]. Both direct (e.g., through psychopharmacological pathway) and indirect (e.g., marital conflicts) effects of alcohol misuse on IPV may explain our results. To further examine which one specifically explains the results among the participants, we need to collect information regarding whether alcohol misuse occurs immediately prior to the IPV, which was not available in the present study. Besides the two possible pathways, it has been reported that antisocial personalities may also explain the association [23].

The observed association is of concern given the high level of alcohol misuse among both Russian men and women. Although the prevalence of alcohol misuse observed in the present study is lower than that observed in another study conducted in Arkhangelsk, Russia by using AUDIT criteria [2], it remains high compared to prevalence found in the United States [24,25]. This finding suggests that efforts to reduce IPV in Russia shall consider the effects of alcohol misuse. Although disagreement exists regarding the question of whether there is enough information to act on IPV through alcohol reduction [26], some studies have provided evidence supporting such interventions [22,27]. In a study conducted among 301 male alcoholic patients from two alcoholism clinics in the northeastern United States, an integrated treatment approach for alcoholism consisting of 26 planned sessions decreased the prevalence rate of IPV by more than 50% [22]. The sessions included an intake assessment, a physical examination, eight individual therapy sessions, and 16 group therapy sessions over a 12-week period. It has also been reported that some brief counseling interventions could cost-effectively reduce alcohol misuse [28]. More studies should be conducted in order to determine whether such brief counseling interventions on alcohol misuse can reduce IPV, particularly in countries such as Russia that feature a high level of alcohol consumption and a cultural IPV tolerance [14].

A striking finding of this study is that participants who usually drank on the street or at parks were more likely to perpetrate IPV than those without this preference. It should be noted that this association was observed after controlling for important confounders in the present study such as alcohol misuse, marital status, and monthly income. Previous studies have reported that drinking at bars is independently associated with violence [6,29]. For example, a study that used data from the 1984, 1995, and 2005 U.S. National Alcohol Surveys implied that bar drinkers tended to report more arguments and fighting than home drinkers, controlling for potential confounders such as the overall volume of alcohol consumed and the frequency of heavy drinking [29]. The authors argued that this association might result from the clustering of intoxicated people, as well as from fewer restrictions on social behaviors due to the looser norms in bars [29]. It is possible that the fewer restrictions theory may also explain our finding, since drinking at streets or parks may be less restrictive even than drinking at bars. If this is the case, policies restricting people drinking on the streets or at parks may effectively reduce IPV.

An alternative explanation for the association between drinking on the streets or at parks and IPV is that drinking on the streets or at parks may have a distal influence on IPV. That is to say, certain factors underlying this drinking preference may be a risk for IPV. For example, those who usually drink on the street or in parks may share similar cultural environments in which IPV may be more common compared to those who drink at other locations. It has been reported that in some cultures, drinking may serve as an excuse for IPV [6]. If this is the case, it is more important to identify these people rather than restrict them from drinking on the street or in parks. Longitudinal studies that analyze contextual drinking patterns of individuals in conjunction with patters of groups of individuals would help us gain a better understanding of contextual alcohol intake. Such understanding is important for the development of appropriate IPV prevention strategies.

The study has several limitations. First, the prevalence of IPV in the past three months might be underestimated because self-reported IPV measurements are likely to be affected by a social desirability bias. To minimize such bias, self-administered questionnaires were used to collect information. Second, the method used to identify significant drinking contexts may increase the chances of detecting a significant association because a series of logistic regression models were conducted to examine the relationship between different drinking contexts and IPV. Third, the confidence interval for the association between drinking context and IPV is relatively wide; this suggests that future studies with larger sample sizes may be needed to better understand such correlations. Fourth, the results may not be generalized to other populations or clinical settings. Fifth, this is a cross-sectional study and thus a causal relationship cannot be established.

## Conclusion

The present results demonstrate that both alcohol misuse and certain drinking contexts (i.e., drinking on the street or in parks) are associated with IPV. Future research needs to investigate whether drinking on the street or in parks represents contexts that facilitate IPV or whether this depicts a sub-group of individuals at risk for IPV. IPV prevention initiatives might benefit from the reduction of alcohol misuse. This might include targeting contextual levels of drinking or addressing factors that are associated with drinking on the street or in parks.

## Competing interests

The authors declare that they have no competing interests.

## Authors' contributions

NA: main contributor to conception, statistic analyses and writing. WZ: contributed to data analysis, interpretation, and drafting this manuscript. AS, contributed to data acquisition, coordination and management, as well as quality control and interpretation of data. RS, contributed to data acquisition, coordination and management, as well as quality control and interpretation of data. AK, contributed to conception and review of the manuscript. TK, contributed to data acquisition, quality control and interpretation and management of data. All authors read and approved the final manuscript.

## Pre-publication history

The pre-publication history for this paper can be accessed here:

http://www.biomedcentral.com/1471-2458/11/629/prepub
